# Molecular Dynamics Simulation Reveals Specific Interaction Sites between Scorpion Toxins and K_v_1.2 Channel: Implications for Design of Highly Selective Drugs

**DOI:** 10.3390/toxins9110354

**Published:** 2017-11-01

**Authors:** Shouli Yuan, Bin Gao, Shunyi Zhu

**Affiliations:** 1 Group of Peptide Biology and Evolution, State Key Laboratory of Integrated Management of Pest Insects and Rodents, Institute of Zoology, Chinese Academy of Sciences, Beijing 100101, China; yuanshouli123@163.com (S.Y.); gaob@ioz.ac.cn (B.G.); 2 College of Resources and Environment, University of Chinese Academy of Sciences, Beijing 100049, China

**Keywords:** K_v_1.2 channel, scorpion toxin, molecular dynamics simulation

## Abstract

The K_v_1.2 channel plays an important role in the maintenance of resting membrane potential and the regulation of the cellular excitability of neurons, whose silencing or mutations can elicit neuropathic pain or neurological diseases (e.g., epilepsy and ataxia). Scorpion venom contains a variety of peptide toxins targeting the pore region of this channel. Despite a large amount of structural and functional data currently available, their detailed interaction modes are poorly understood. In this work, we choose four K_v_1.2-targeted scorpion toxins (Margatoxin, Agitoxin-2, OsK-1, and Mesomartoxin) to construct their complexes with K_v_1.2 based on the experimental structure of ChTx-K_v_1.2. Molecular dynamics simulation of these complexes lead to the identification of hydrophobic patches, hydrogen-bonds, and salt bridges as three essential forces mediating the interactions between this channel and the toxins, in which four K_v_1.2-specific interacting amino acids (D353, Q358, V381, and T383) are identified for the first time. This discovery might help design highly selective K_v_1.2-channel inhibitors by altering amino acids of these toxins binding to the four channel residues. Finally, our results provide new evidence in favor of an induced fit model between scorpion toxins and K^+^ channel interactions.

## 1. Introduction

The voltage-gated K^+^ channel K_v_1.2, encoded by *KCNA2*, is a transmembrane protein that is composed of four identical α-subunits, with each subunit having six transmembrane segments (S1–S6) and a membrane reentering P-loop. As shown by their experimental structure [1], S1–S4 form a voltage-sensor domain (VSD) and S5–S6 constitute a pore that selectively passes K^+^ ions [2]. K_v_1.2 plays an important role in maintaining the resting membrane potential that enables efficient neuronal repolarization following an action potential [3]. Therefore, its loss or mutation will cause some neurogenic diseases, such as ataxia, myoclonic epilepsy, and premature death [4,5,6,7].

**Figure 1:**
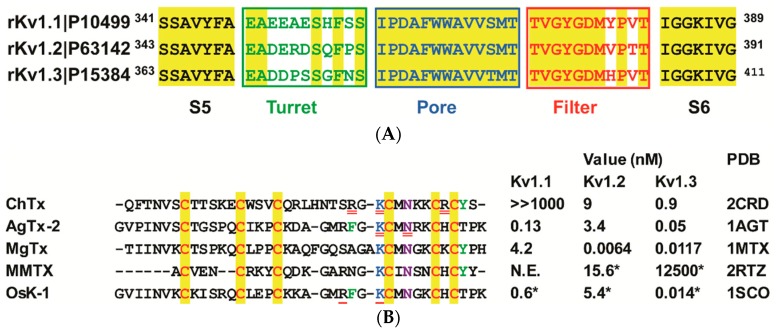
Scorpion toxins and K^+^ channels. (**A**) The rK_v_1.1-rK_v_1.3 pore region sequences. Amino acids conserved across the alignment are marked in yellow, and turret, pore helix, and filter are colored in green, blue, and red, respectively; (**B**) The α-KTxs studied in this work. Their functional sites identified by mutational experiments are highlighted with a double underline. New interaction sites predicted by reported molecular dynamics (MD) simulation data are underlined once. For the affinity of each toxin, Kd (dissociation constant), Ki (equilibrium constant), or IC_50_ (half maximal inhibitory concentration) (asterisks) are shown in nanomole (nM) [8,13,14,15,16,17].

Given its key physiological function, K_v_1.2 is frequently selected as a target by a diversity of venomous animals. In scorpions, at least six families of K^+^ channel toxins (α-KTxs, β-KTxs, δ-KTxs, κ-KTxs, λ-KTxs, and ε-KTxs) are identified [2,8,9,10,11,12], in which α-KTxs are the largest source targeting this channel. These α-KTxs impair the K^+^ channel functions by blockage of their pore region. Due to high sequence similarity in this region between K_v_1.2 and its two paralogs (i.e., K_v_1.1 and K_v_1.3) (Figure 1A), toxins are not able to distinguish the three channel subtypes, resulting in undesired side effects when assayed in vivo. Hence, it remains a great challenge to improve the K_v_1.2 selectivity of these natural toxins via engineering modification in the absence of detailed data about the toxin-channel interactions.

There is only one experimental toxin-channel complex (ChTx-K_v_1.2) available currently [18], which hampers a detailed comparative study to draw commonality and difference among complexes. An alternative approach is to employ computative technology to solve this question. Several popular methods include homology modeling, Brownian dynamics, molecular docking, and molecular dynamics simulation [19]. Molecular dynamics simulation is a powerful tool in predicting the structures of toxin-channel complexes. Some toxin-K_v_1.2 complexes were reported, which were constructed with molecular dynamics simulation, such as maurotoxin-K_v_1.2 [20,21]. However, the comparison of the mechanism among these K_v_1.2 inhibitors is lacking.

In this work, we employed molecular dynamics (MD) simulation to study the interactions of four α-KTxs (Margatoxin (abbreviated as MgTx), Agitoxin-2 (abbreviated as AgTx-2), OsK-1, and Mesomartoxin (abbreviated as MMTX) with the pore of K_v_1.2. These toxins all bind to the channel with high affinity [8,13,14] (Figure 1B). However, as mentioned above, they are also ligands of K_v_1.1 and K_v_1.3, with the exception of MMTX, which lacks effect on K_v_1.1. Our MD simulation data reveals for the first time four K_v_1.2-specific amino acids that are involved in direct interactions with these toxins. This finding thus provides a structural basis for their K_v_1.2 blocking activity and might help design new K_v_1.2-targeted peptide drugs with an improved channel subtype selectivity.

## 2. Results

### 2.1. The Channel Selectivity of Four Scorpion Toxins Analyzed

All the four toxins fold into the cysteine stabilized α-helix/β-sheet (CSα/β) structure with a functional dyad comprising a conserved lysine and an aromatic amino acid in a distance of 5–7 Å (Figure 1B). These toxins reversibly block K^+^ channels by interacting at the external pore of the channel protein. Of them, MgTx is a 39 amino acids peptide isolated from the venom of the scorpion *Centruroides margaritatus*, which blocks K_v_1.2 and K_v_1.3 at picomolar concentrations and K_v_1.1 at nanomolar concentrations without detectable effect on other types of K^+^ channels, such as K_v_1.4–K_v_1.7 and the insect *Shaker* K^+^ channel [15]. K28 of MgTx is responsible for blocking K^+^ channels [22]. AgTx-2 is a 38 amino acids peptide isolated from the venom of *Leiurus quinquestriatus hebraeus*, which reversibly inhibits K_v_1.1 to K_v_1.3 with Kd values of 0.13 nM, 3.4 nM, and 0.05 nM, respectively [8,16]. Its functional residues include K27 and N30 [17]. OsK-1 is a 38 amino acids peptide isolated from the venom of *Orthochirus scrobiculosus*, which blocks K_v_1.1 to K_v_1.3 with IC_50_ values of 0.6 nM, 5.4 nM, and 0.014 nM, respectively [14]. E16K and/or K20D mutations of OsK-1 show an increased potency on K_v_1.3 channel but do not change the effect on K_v_1.2 [14]. MMTX is a 29 amino acids peptide isolated from *Mesobuthus martensii* that exerts a strong inhibitory effect on rK_v_1.2 (IC_50_ = 15.6 nM) and weak effect on rK_v_1.3 (IC_50_ = 12.5 μM) without affecting K_v_1.1, even at 50 μM [13].

### 2.2. Modeling of Toxin-K_v_1.2 Complexes

**Figure 2:**
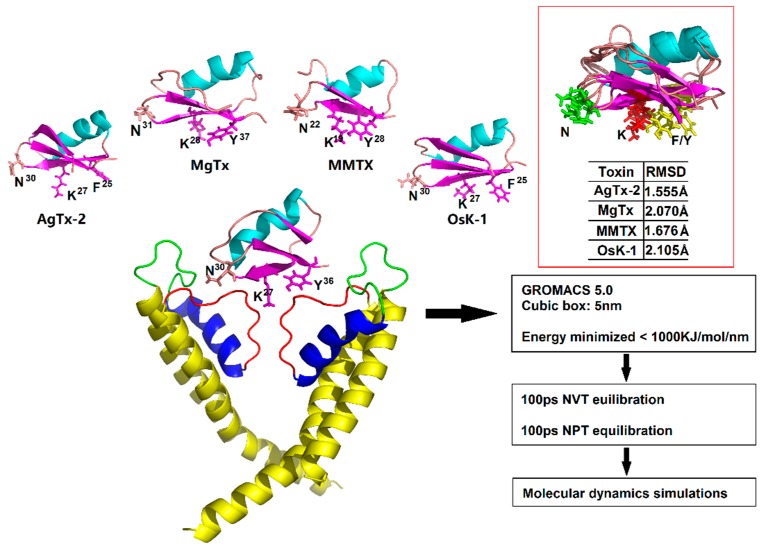
Construction of toxin-channel complex for MD simulation analysis. Three experimentally determined functional sites of α-KTxs are shown as sticks. Superimposed structure of these five toxins are emphasized by red box and root-mean-square deviations (RMSDs) between these four toxins and ChTx are listed below the structure.

ChTx (also named CTX) is the most thoroughly studied scorpion K^+^ channel toxin isolated from the venom of *Leiurus quinquestriatus hebraeus*, which inhibits K_v_1.2 and K_v_1.3 channels with nanomolar affinity by several crucial functional residues, such as R25, K27, and R34 [8,23]. K27 of ChTx is a key residue for blocking the pore region of the K^+^ channel [24]. Superimposition of the four toxins to ChTx reveals root-mean-square deviations (RMSDs) of <2.5 Å in their Cα atoms (Figure 2), indicating that they are quite similar in structure. Importantly they all contain evolutionarily conserved functional motifs, K27 and N30 (numbered according to ChTx), which have been comfirmed to directly interact with the pore region of K_v_ channels [25,26]. Supported by these observations, we assume that all these five toxins inhibit K_v_1.2 in a similar manner. To investigate the detailed interactions between the toxins and K_v_1.2, we constructed their complexes based on the experimental structure of ChTx-K_v_1.2 via molecular replacement and energy minimization.

### 2.3. Conformational Changes Induced by Toxin-Channel Interaction

To recognize the conformation change of toxins and K_v_1.2 channel after combining each other, we play molecular dynamic simulations of four toxins without channel and with sole K_v_1.2 channel. Subsequently, we compared their conformation changes.

**Figure 3:**
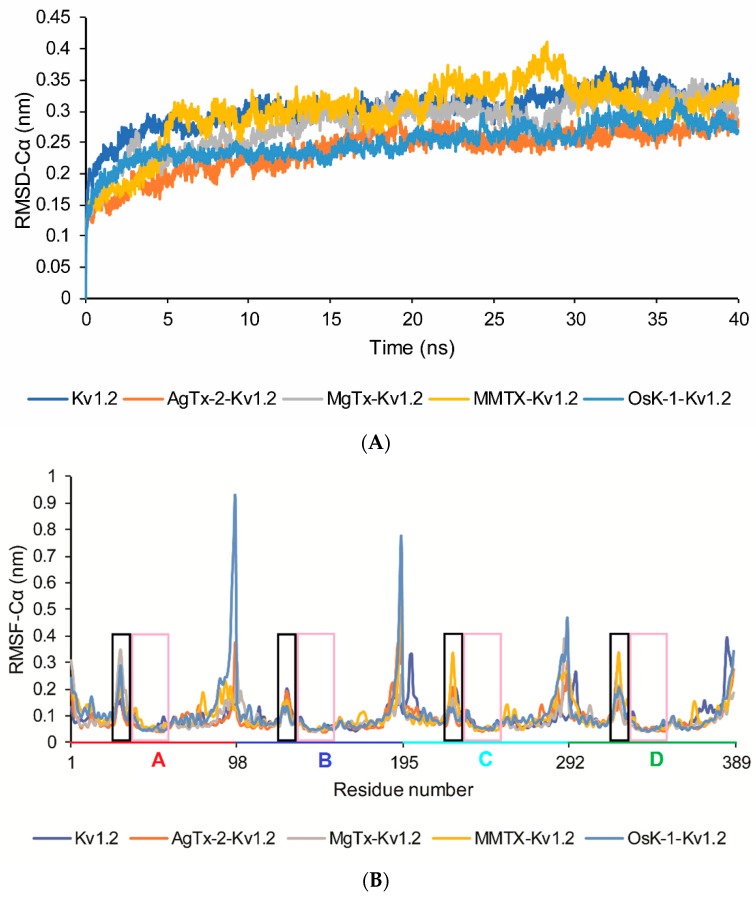

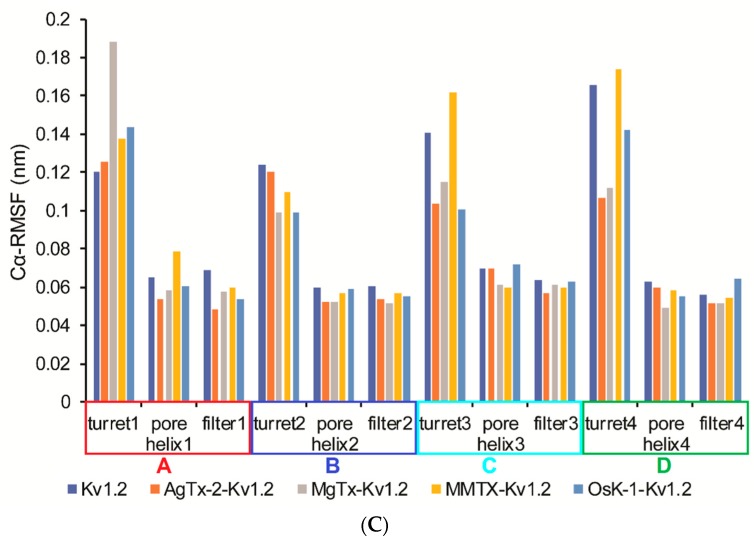
Structural flexibilities of four toxin-K_v_1.2 complexes. (**A**) RMSDs of four toxin-K_v_1.2 pore region complexes; (**B**) root-mean-square fluctuations (RMSFs) of the Cα atoms of K_v_1.2 pore region in these four complexes from 15 ns to 40 ns. A–D indicate four different chains in K_v_1.2. The range of A–D chains’ pore region residue number is marked by red, blue, light blue, and green string, respectively. Turret regions are outlined by black rectangular boxes and pore helix and filter regions are outlined by pink rectangular boxes; (**C**) average Cα-RMSF of K_v_1.2 pore region in sole K_v_1.2 and these four complexes from 15 ns to 40 ns. Turret, pore helix, and filter in *x*-coordinate represent the turret, pore helix, and filter region of K_v_1.2 channel. A–D indicate four different chains in K_v_1.2.

In our molecular dynamic simulations, the equilibrated conditions of four toxin-channel complexes were established in terms of their RMSDs, residue Cα fluctuations during 40 ns time span of simulation. These systems reached equilibrium after 15 ns (Figure 3A). Simultaneously, we calculated the average Cα root-mean-square fluctuations (RMSFs) of all complexes of the K_v_1.2 pore region. From the RMSF data, it is clear that K_v_1.2 turret is the most flexible region besides the *N*-, *C*-terminal (Figure 3B,C). Therefore, we proposed that K_v_1.2 channel interacts with different scorpion toxins, mainly by modulating their turret region.

**Figure 4:**
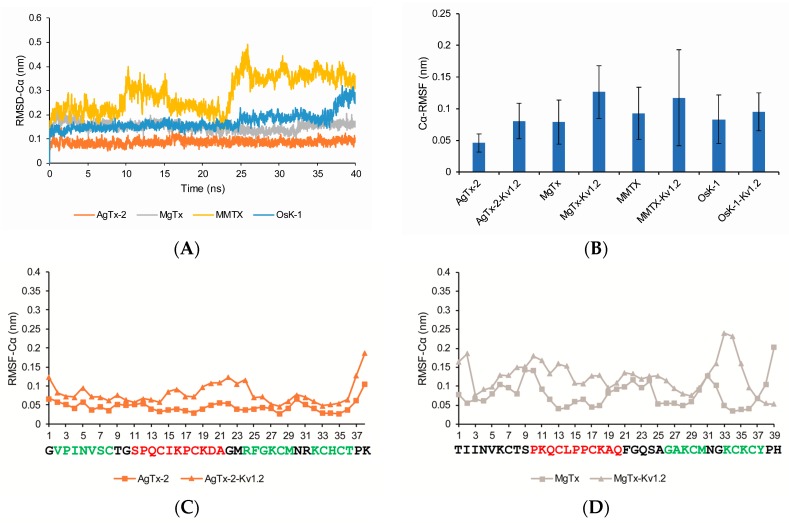

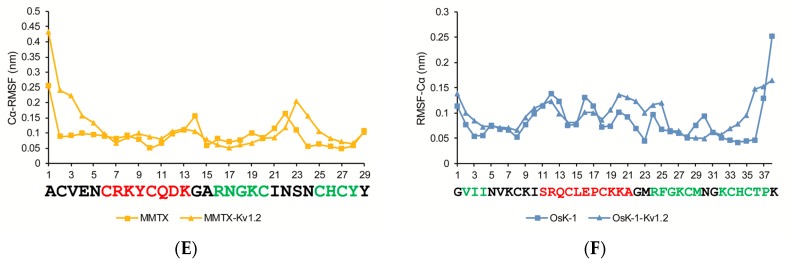
Structural flexibilities of four toxins. (**A**) RMSDs; (**B**) average RMSF; (**C**–**F**) RMSFs of the Cα atoms of four toxins. Toxins’ sequences are written under x-coordinate and their α-helical and β-fold are colored in red and green, respectively.

The equilibrated conditions of four toxins were established in terms of their RMSDs and residue Cα wise fluctuations during 40 ns time span of simulation. MgTx and AgTx-2 reached equilibrium after 5 ns and MMTX reached equilibrium after 27 ns (Figure 4A). The system equilibrium stage of OsK-1 is from 5 ns to 35 ns (Figure 4A). We calculated the average Cα root-mean-square fluctuations (RMSFs) of their system equilibrium phase (Figure 4B). Without a doubt, the results show that these four toxins are very rigid, because α-KTxs obtain six conservative cysteines which form three intermolecular disulfide bonds. Due to their stable structure, they are developed into protein scaffolds. For example, Vita et al. designed a metal binding activity on ChTx [27]. After combining with K_v_1.2, their structure mildly adjusted. OsK-1 and AgTx-2 have little change after combining with K_v_1.2, which indicate that its interaction mechanism with K_v_1.2 is similar to ChTx (Figure 4C,F). MgTx shows the increased flexibility of α-helix and γ-core region (the last two β-folds and the turn region between them) (Figure 4D). MMTX shows the increased flexibility of N-terminal (Figure 4E). 

### 2.4. The Interactions of α-KTxs with K_v_1.2

**Figure 5:**
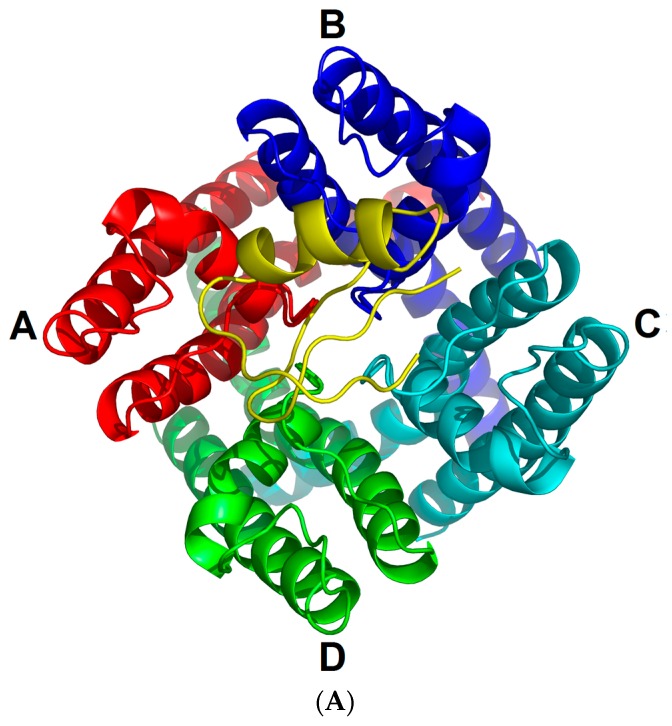

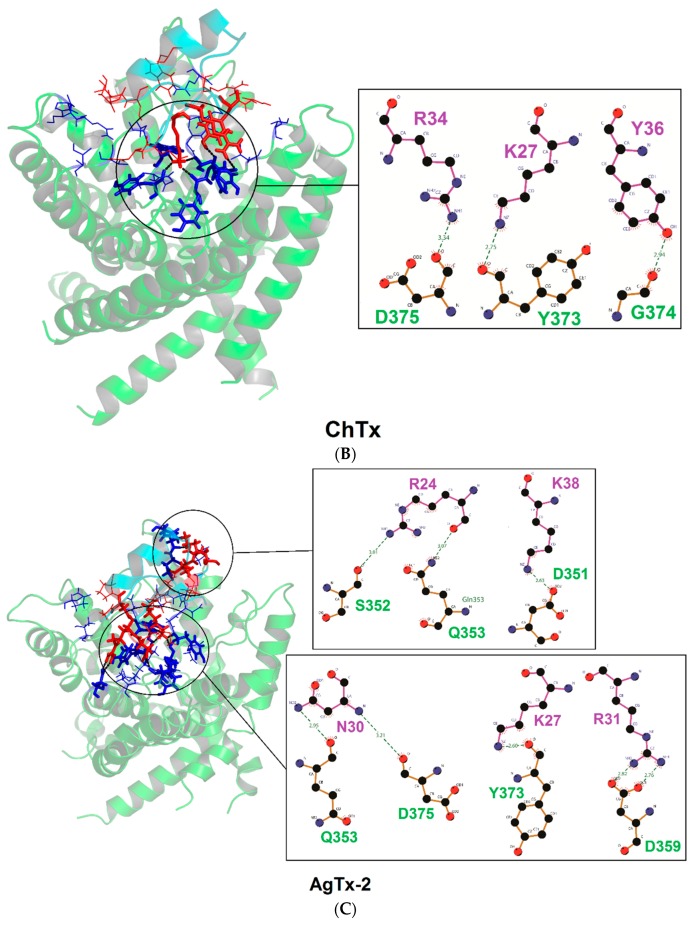

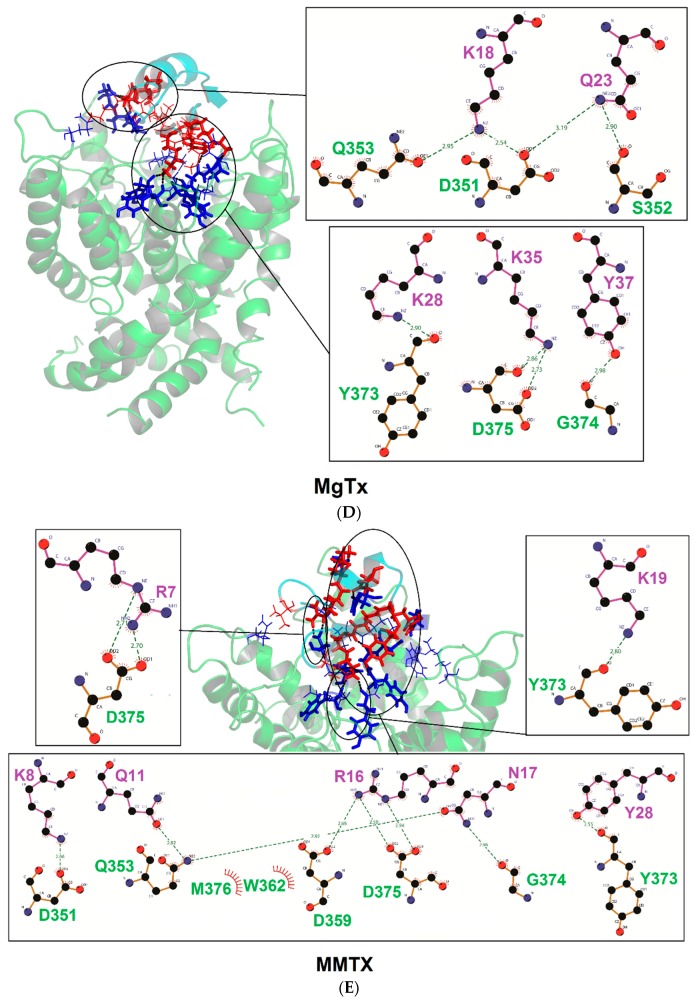

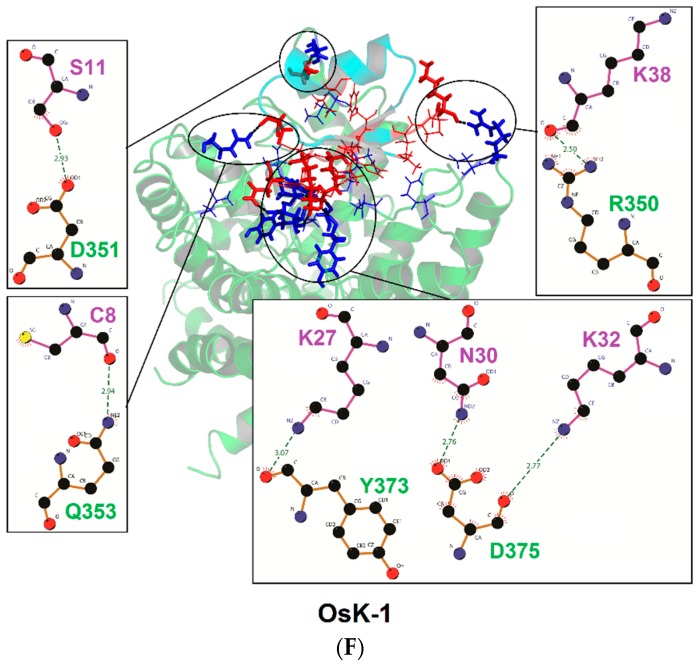
Interactions between toxins and K_v_1.2. (**A**) A–D chains of K_v_1.2 pore region; (**B**–**F**) showing the interaction residues of toxins and channel. H-bonds are shown in green and hydrophobic interactions in red. Interaction sites of toxins are highlighted in red and sites of the channel in blue. Sites involved in hydrophobic interactions are shown as lines and sticks in H-bonds or salt bridges.

**Figure 6:**
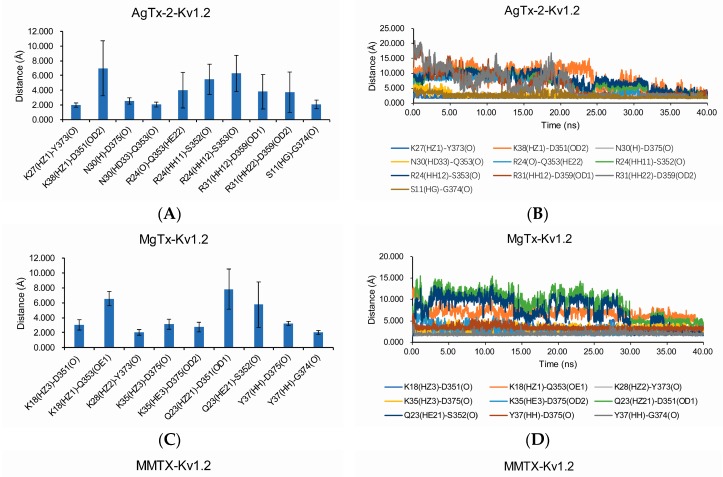

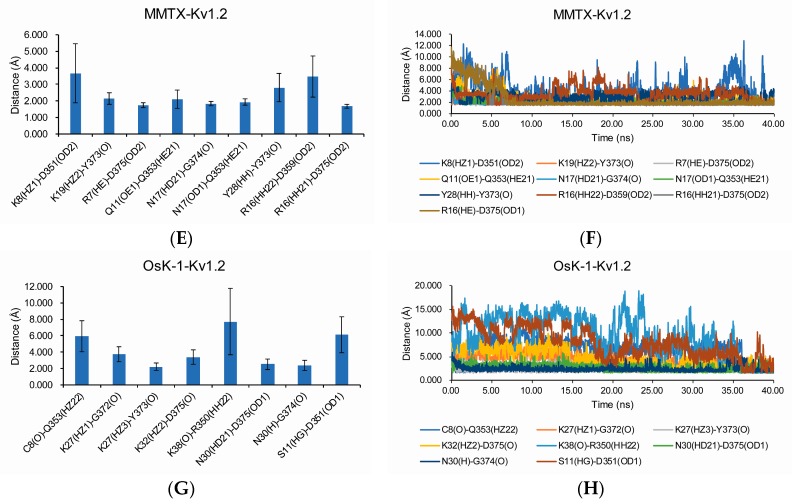
Hydrogen bonds and salt bridges distances of toxin-K_v_1.2 complexes. (**A**,**C**,**E**,**G**) showing the average distances of hydrogen bonds and salt bridges from 15 ns to 40 ns. (**B**,**D**,**F**,**H**) showing the changes of hydrogen bonds and salt bridges distances between toxins and K_v_1.2 channel in 40 ns.

Using LigPlot^+^ software, we analyzed the constructed complexes, together with ChTX-K_v_1.2 for comparison purposes. Their detailed interactions are shown in Figure 5 and Table 1. To ensure these predicted hydrogen bonds and salt bridge are reliable, we calculated these bonds’ distances in 40 ns (Figure 6).

Our complexes of K_v_1.2 and toxins are consistent with the current experimental data. AgTx-2 have been reported that K27 and N30 are critical for binding affinity toward Shaker K^+^ channel [17]. In our AgTx-2-K_v_1.2 model, K27 inserts into pore region and forms H-bond with Y373, and N30 forms H-bond with Q353 and D375 (Figure 5C). Mutation experiments showed that MMTX interact with rK_v_1.2 V379. In our MMTX-K_v_1.2 model, R7 of MMTX interacts with V379 through hydrophobic contact (Table 1) [13]. The OsK-1-K_v_1.2 model suggest that E16 and K20 of OsK-1 does not interact with K_v_1.2. It accords that E16K and/or K20D mutations of OsK-1 do not change the effect on K_v_1.2 (Table 1) [14].

**Figure 7:**
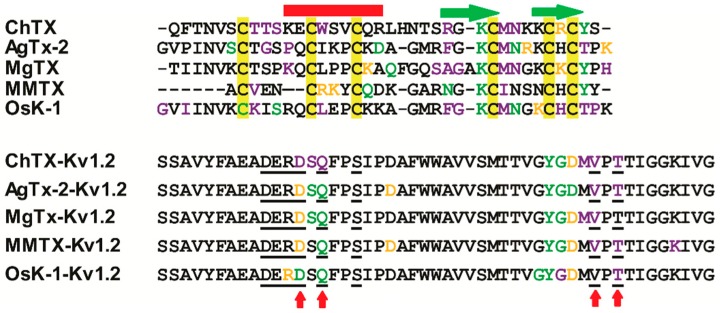
Interaction sites between toxins and K_v_1.2. Sites participating in salt bridges, H-bonds, and hydrophobic effect are highlighted in golden, green, and purple, respectively. Four variable channel sites implicated in the interaction with α-KTxs are labeled with red arrows.

As expected, these five toxins all present some commonalities in interacting with K_v_1.2. They depend on hydrophobic contacts, hydrogen-bonds (H-bonds), and salt bridges to stay close to the channel where K27 inserts into the pore and forms H-bonds with Y373 (unless otherwise stated, all toxins and the channel are numbered according to the ChTX-K_v_1.2 complex [18]). An aromatic amino acid (F or Y) belonging to the functional dyad interacts with D375 or Y373. With the exception of MMTX, all the toxins use M29 and N30 to contact G374 and D375 in the channel filter region through the van der Waals force. Furthermore, in our AgTx-2-K_v_1.2 model, N30 also forms H-bond with Q353 and D375. These observations fully confirm the functional importance of K27 and N30 in this toxin previously obtained by mutational analysis [17]. The turn region preceding the α-helix of the toxins contacts the turret residue S353 and the filter D375/V377/T379 via a hydrophobic interaction force. We also observed that the basic amino acids located at the last β-strand of the toxins, except MMTX, form salt bridges with the acidic Asp of K_v_1.2. R34 of ChTx, K35 of MgTx, and K32 of OsK-1 form salt bridges with D359 of K_v_1.2 and R31 of AgTx-2 form salt bridges with D359 of K_v_1.2. These toxins have conservative six cysteines that hardly participate in the interaction with K_v_1.2, but they are related to structural stabilization [28]. Compared with other toxins, MMTX forms more hydrogen bonds and salt bridges with the channel. Especially, due to its shorter N-terminus, this toxin can enter more deeply into the channel pore to form salt bridges between K8 or R16 and the residues derived from the chain B of the channel during MD simulation. These noncovalent interactions could facilitate the formation of a more stable toxin-channel complex [29]. In our MMTX-K_v_1.2 model, R7 of the toxin interacts with V377 of the channel through hydrophobic contact, in line with the mutational experiments that highlighted this channel residue as a target site of MMTX [13]. Taken together, our MD simulation results provide support for the toxins’ binding sites mainly locating at the K_v_1.2 turret and filter region and are thus a reasonable explanation for the lack of channel subtype selectivity in these toxins given that in these regions most sites are highly conserved among K_v_1.1–K_v_1.3 (Figure 7).

### 2.5. K_v_1.2-Specific Amino Acids

**Figure 8:**
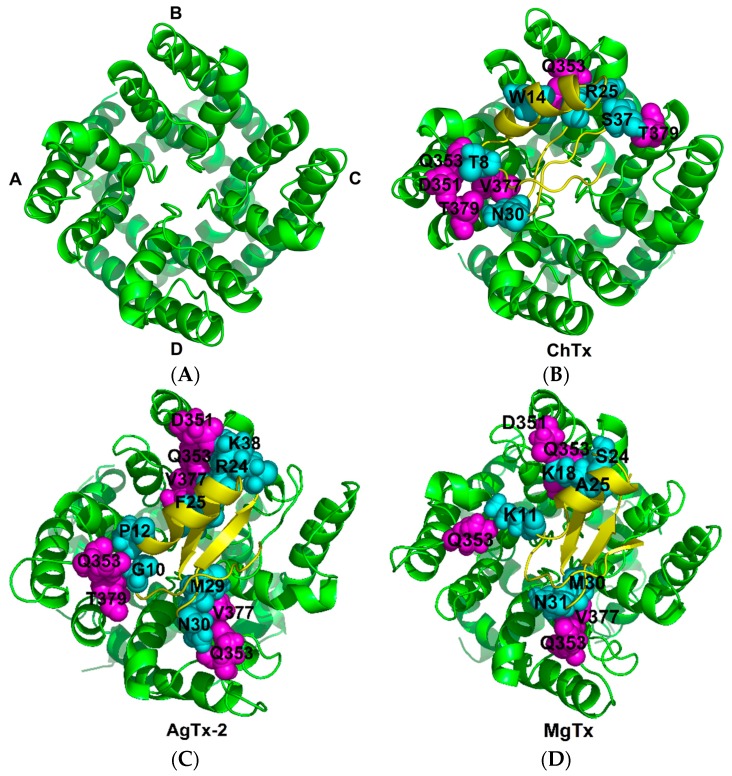

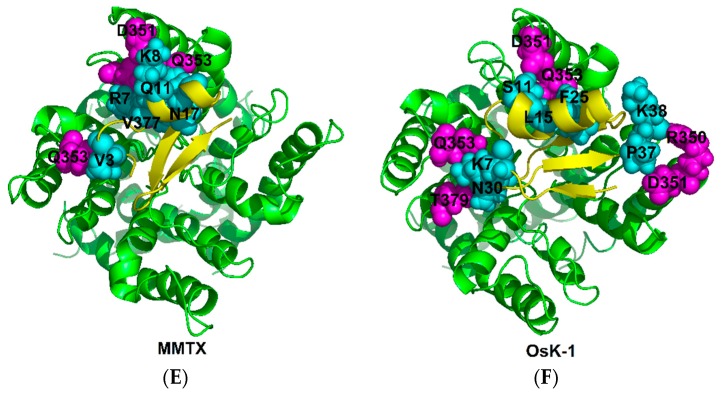
K_v_1.2 specific amino acids involved in the interactions with the toxins. (**A**) A–D chains of K_v_1.2 channels pore region; (**B**–**F**) interaction sites of toxins are highlighted in cyan; unconservative sites in the channel are highlighted in magentas. Amino acids at the interface between a toxin and the channel are shown as spheres.

More importantly, through analysis of the interaction between these four toxins and K_v_1.2 channel, we observed four K_v_1.2-specific amino acids (D355, Q358, V381, and T383 of rK_v_1.2) that can interact with the toxins (Figure 7 and Figure 8). In these interactions, D355 and Q358 form hydrogen bonds or salt bridges whereas V381 and T383 form hydrophobic interactions with the toxins. This observation could help answer the differential affinity of these toxins towards K_v_1.1 to K_v_1.3. For example, the preferred inhibition of K_v_1.2 and K_v_1.3 over K_v_1.1 by Aam-KTX is explained by the variation at site 355 (corresponding to the K_v_1.2-specific amino acid Q358). In K_v_1.1, this site is occupied by a larger His that might hamper the toxin’s entry into its pore vestibule [30]. In addition, site 381(Val) has been proposed as a main determinant of MMTX’s selectivity towards K_v_1.2 over K_v_1.1 [13], in agreement with our MD simulation data. In the MgTx-K_v_1.2 complex, K18 from the toxin forms an H-bond with Q358 from the channel. The corresponding amino acid at this site is a His in K_v_1.3. Because histidine is the same kind of charge with lysine, their repulsion is adverse to the formation of an H-bond. For K_v_1.1, a glycine occupies this position and its shorter side chain also hampers the formation of the H-bond. These observations could account for the differential in affinity of MgTx to these three channels (Figure 1A). Relative to other toxins analyzed here, MMTX possess a shorter *N*-terminus and is the only one without effect on K_v_1.1. In our complex, its K8 forms salt bridges with D355, whereas sites Q11 and N17 form H-bonds with Q358. These extensive contacts likely provide a structural basis for its high activity on K_v_1.2.

## 3. Discussion

It is long known that the evolutionary conservation of the pore region among K_v_1.1 to K_v_1.3 poses a challenge to animal toxin-based drug design. To face this challenge, three kinds of protein engineering technologies have been explored to improve their selectivity: (1) Site-directed mutation. Using this technology, several highly selective scorpion toxins against K_v_1.3 have been obtained [31]; (2) Phage display technology. This technology led to the discovery of Mokatoxin-1, an engineering peptide that blocks K_v_1.3 at the nanomolar level without effect on K_v_1.1, K_v_1.2 and K_Ca_1.1 [8]; (3) MD simulation. This technology can guide molecular design based on the structural features of peptides [32].

In this work, through using MD simulation technology, we identified four K_v_1.2-specific amino acids that are involved in the interactions with different scorpion toxins (Figure 7). These K_v_ specific sites might be the reason that toxins have different targets. Conservative sites of α-KTxs interact with conservative sites of K_v_ channels. For example, K27 of α-KTxs insert into K_v_ channel pore region, form H-bonds with Y373, and inhibit K^+^ pass. TVGYG motif is conservative among eukaryotic K_v_ channel and important for binding K^+^ [19]. Nevertheless, unconservative sites of α-KTxs interact with K_v_ specific sites which determine the selectivity of different toxins. Like scorpion Na+ channel, α-toxins has a common bipartite bioactive surface: (1) Conserved core-domain are associated with toxin potency, which interact with domain IV S1–S2 and S3–S4 of Na_v_ channels; (2) Variable NC-domain dictate toxin’s selectivity, which interact with domain I S5–S6 of Na_v_ channels [33]. According to these facts, we proposed that the evolution of toxins has a common rule: conservative domain ensures their potency, which interacts with receptor and the variable domain convenient to adjusting their targets.

Furthermore, in combination with the MD simulation data presented here, we proposed a concept of “triplet-motif” for channel blockade. This motif is composed of the dyad comprising a lysine located at the first β-strand and an aromatic amino acid in a distance of 5–7 Å and an Asn in the loop linking the first and second β-strands, two residues downstream from the Lys (i.e., LysCysXaaAsn. Xaa, any amino acids). The location of these three functional sites (dyad-motif and N30) is shown on Figure 2. The inclusion of this residue into the toxins’ functional motif is based on the following considerations: (1) This residue is an evolutionarily highly conserved amino acid that belongs to the scorpion toxin signature (STS), comprising Cys…CysXaaXaaXaaCys…LysCysXaaAsn…CysXaaCys; (2) Its functional significance has been highlighted in some toxins (e.g., AgTx-2, navitoxin, etc.) [17]; (3) In an NMR-based complex of KTX and KcsA-K_v_1.3, N30 is close to D64 of KcsA-K_v_1.3 [25]; (4) In our dynamics structures, this residue in AgTx-2, MgTx, and OsK-1 forms H-bonds or hydrophobic interactions with K_v_1.2.

The molecular mechanism of interaction between scorpion toxins and K_v_ channels has always been controversial. There are currently two hypotheses: (1) The induced-fit model. Using high-resolution solid-state NMR spectroscopy, Lange et al. observed chemical shifts occurring in some residues of kaliotoxin (KTx) and KcsA-K_v_1.3 during their interactions. For the channel, significant chemical shifts appeared in the pore helix and the selectivity filter [25]. Because of these observations, they thought that the toxin binds to the channel in an induced fit manner; (2) The lock-and-key model. This model is based on the consideration of rigidity of the K^+^ channel pore region [34], evidenced by a crystal structure study that resolved the structures of K_v_1.2–K_v_2.1 in complex with ChTx and the channel alone. When the complex was superposed onto the K_v_1.2–K_v_2.1 structure, no discernible structural changes were observed in the channel [18]. Therefore, they proposed that scorpion toxins bind to K^+^ channels in a lock and key manner. According to our molecular dynamics simulation result, the turret region of K_v_1.2 is the most flexible region. Therefore, we proposed that K_v_ channels interact with different scorpion toxin mainly by modulating their turret region. Consider existing experimental data, different sequence and structure toxins block K_v_1.2 though pore region inhibition and these toxins bind to more than one channel [2,35,36,37,38]. Our result and these experimental data all support induced fit model.

## 4. Conclusions

By MD simulation analysis combined with previous experimental data, we reveal a common mode adopted by scorpion K^+^ channel toxins in binding to the channels, in which the conserved and variable toxin functional residues seem to interact with the conserved and subtype-specific channel residues, respectively. This finding provides new candidate sites in the toxins for mutations to improve their selectivity towards a specific channel subtype.

## 5. Materials and Methods

### 5.1. Atomic Coordinates and K_v_1.2-Toxin Complexes

Atomic coordinates of ChTx-K_v_1.2-K_v_2.1 chimera (PDB: 4JTA), AgTx-2 (PDB: 1AGT), MgTx (PDB: 1MTX), MMTX (PDB: 2CRD), and OSK1 (PDB: 1SCO) were retrieved from the Protein Data Bank [39]. All toxin-K_v_1.2 complex structures studied here were built by the Swiss PDB Viewer software (http://spdbv.vital-it.ch/), in which ChTx-K_v_1.2-K_v_2.1 chimera was used as template. These four toxins were aligned onto the ChTx-K_v_1.2-K_v_2.1 chimera and then deleted ChTx to build all toxin-K_v_1.2 complexes. The channel pore region (residues 321 to 417) were used in this study.

About building the complexes of toxins and channels, some groups choose ZDOCK or HADDOCK molecular docking software to obtain the complexes [20,40,41]. Since the structure of ChTx and K_v_1.2–2.1 chimera resolved, several groups use this resolved structure to build scorpion toxins and K^+^ channel complexes [42,43]. Nekrasova et al. performed homology modeling of K_v_1.6-toxin complexes instead of molecular docking to obtain a more reliable model [43]. Therefore, we choose ChTx and K_v_1.2–2.1 chimera to build our toxin-channel complexes.

### 5.2. Molecular Dynamics Simulation

Molecular dynamics simulations were performed using Gromacs 5.0.1, in which all-atom OPLS force field was chosen [44,45]. The complex was solved with SPC water [46] and was immersed in a cubic box extending to at least 5 nm of the solvent on all sides. Also, the system was neutralized by K^+^ and Cl^−^. It was energy minimized by using the steepest descent algorithm for 5000 steps, and it made a maximum force of less than 1000 kJ/mol/nm. After energy minimization, the system was equilibrated in a constrained NVT (Number of Particles, Volume, Temperature) and NPT (Number of Particles, Pressure, Temperature) running for 100 ps. NVT equilibration ensured the system be brought to the temperature (300 K) which we wish to simulate, and with which we seek to establish the proper orientation about the protein. After NVT equilibration, we stabilize the pressure of the system under an NPT ensemble. Through NVT and NPT equilibration, it was well-equilibrated at 300 K and 1 bar. Bond length was constrained using the LINCS algorithm [47]. Finally, MD simulations of these complexes were carried out for 40 ns. Trajectories are saved every 10ps for analysis. For the MD simulation, the Verlet cut-off scheme and a Leap-frog integrator with a step size of 2 fs were applied. For temperature coupling, the modified Berendsen thermostat and the Parrinello-Rahman barostat for pressure coupling were used. For long-range electrostatic interaction, the Particle Mesh Ewald method was used. The method of four toxins’ molecular dynamics simulation is similar to the toxin-K_v_1.2 complex. The differences are that the toxins were neutralized by Na^+^ and Cl^−^, and they were immersed in a cubic box extending to at least 1 nm of the solvent on all sides.

The mutagenesis and simulations indicated that the scorpion toxins bind with the extracellular part of the K^+^ channels and the interaction is hardly affected by the membrane and the transmembrane segment of channel [48,49,50,51]. We did not add the membrane into the simulation like the work of other study groups [20,52,53,54,55,56,57,58,59]. Also, many simulation studies on the recognition between scorpion toxins and K^+^ channels without a membrane have achieved good agreements with experimental data [53,58,59]. Discarding the lipid-protein interactions has also contributed to the reduction of the computational burden and the extention of the MD simulation trajectories [19]. Certainly, a transmembrane protein system could be more reliable if we take into account the membrane around the channel.

We did not perform similar MD simulations to toxins-channels K_v_1.1 and K_v_1.3, because the K_v_1.1–K_v_1.3 channel only has eight unconservative sites (Figure 1B). And only K_v_1.2–2.1 chimera structure was resolved. In this work, we aim to obtain the K_v_1.2-toxin complexes and understand the detailed interaction between K_v_1.2 and the toxins. This will help us to design mutations according to these complexes and obtain a K_v_1.2 specific selective toxin.

### 5.3. Analysis of MD Simulation Results

After molecular dynamics simulation, we obtained the last MD simulation frame of the complexes. To analyse whether the protein was stable and close to the experimental structure, we measured the root-mean-square displacement (RMSD) of all the structures of Cα. LigPlot^+^ software (http://www.ebi.ac.uk/thornton-srv/software/LigPlus/) was used to analyze the detailed interactions between the toxin and K_v_1.2 [60]. LigPlot^+^ can analyze the hydrophobic interaction, hydrogen bonds, and salt bridges between toxins and K_v_1.2. To ensure the reliability of predicted hydrogen bonds and salt bridges, we calculated the distances of hydrogen bonds and salt bridges in 40 ns using GROMACS. Pymol (http://www.pymol.org/) was used to prepare all the structural images.

There is no unified standard about choosing which structure to analyse during molecular dynamics simulation. Kohl et al. chose the structure with the highest number of H-bonds between the toxin and the channel [42]. Nekrasova et al. chose 70 trajectory frames to analyse the hydrophobic interaction, the hydrogen, and the ionic bonds of the toxin [43]. Also, Yi et al. analysed the last structure of the toxin-channel structure [20,40]. Too short molecular dynamics simulation will cause proteins to have not enough time to change their conformation [61]. Therefore, we gave 40 ns to stabilize the structure and most toxin-channel dynamic studies only run several nanoseconds. Through calculating the distances of H-bonds and salt bridges, these bonds became stable with the extension of simulation time. As a result, we proposed that the last frame of the toxin-channel complex was reliable.

## Figures and Tables

**Table 1:** Interacting pairs between toxins and K_v_1.2 pore region. Unconservative sites among K_v_1.1–K_v_1.3 pore regions are marked by asterisks (*). Chain number of channel amino acids are labeled in brackets.

Interaction Force Type	ChTx	AgTx-2	MgTx	MMTX	OsK-1
Hydrophobic contacts	T8-Q353(A) * T8-D351(A) * T9-S352(A) S10-D375(A) W14-Q353(B) * R25-M376(B) R25-Q353(B) * M29-D375(D) M29-G374(D) N30-V377(A) * N30-T379(A) * N30-D375(D) Y36-D375(B) S37-T379(C) *	G10-T379(A) * P12-Q353(A) * F25-D375(A) F25-G374(B) F25-V377(B) * F25-D375(B) M29-D375(C) M29-G374(D) M29-V377(D) *	K11-Q353(A) * S24-Q353(B) * A25-Q353(B) * G26-D375(B) M30-G374(D) M30-V377(D) * N31-Q353(D) * Y37-D375(B) H39-M376(B)	V3-Q353(A) * R7-V377(B) * R7-T379(B) * R16-K384(C) I21-G374(D) I21-D375(D) Y28-G374(C)	I3-D375(C) K7-Q353(A) * L15-Q353(B) * F25-Q353(B) * G26-D375(B) K27-G374(B) M29-G374(D) N30-T379(A) * H34-D375(C) P37-D351(C) *
H-bonds	K27-Y373(A) K27-Y373(B) K27-Y373(C) K27-Y373(D) Y36-G374(C)	S11-G374(A) R24-S352(B) R24-Q353(B) * K27-Y373(A) K27-Y373(B) K27-Y373(C) K27-Y373(D) N30-Q353(D) * N30-D375(D)	K18-Q353(B) * Q23-S352(B) K28-Y373(A) K28-Y373(B) K28-Y373(C) K28-Y373(D) Y37-G374(C)	Q11-Q353(B) * N17-Q353(B) * N17-G374(B) K19-Y373(A) K19-Y373(C) K19-Y373(D) Y28-Y373(B)	C8-Q353(A) * S11-D351(B) * K27-Y373(A) K27-G372(B) K27-Y373(C) K27-Y373(D) N30-G374(A) N30-D375(D)
Salt bridges	R34-D375(C)	R31-D359(D) K38-D351(B) *	K18-D351(B) * K35-D375(C)	R7-D375(A) K8-D351(B) * R16-D359(B) R16-D375(B)	K32-D375(D) K38-R350(C) *
